# Mapping patients’ experiences from initial symptoms to gout diagnosis: a qualitative exploration

**DOI:** 10.1136/bmjopen-2015-008323

**Published:** 2015-09-14

**Authors:** Jennifer Liddle, Edward Roddy, Christian D Mallen, Samantha L Hider, Suman Prinjha, Sue Ziebland, Jane C Richardson

**Affiliations:** 1 Research Institute for Primary Care and Health Sciences, Keele University, Keele, UK; 2 Nuffield Department of Primary Care Health Sciences, Oxford University, Oxford, UK

**Keywords:** PRIMARY CARE, QUALITATIVE RESEARCH, Gout, Patient experiences, Diagnosis

## Abstract

**Objective:**

To explore patients’ experiences from initial symptoms to receiving a diagnosis of gout.

**Design:**

Data from in-depth semistructured interviews were used to construct themes to describe key features of patients’ experiences of gout diagnosis.

**Participants and setting:**

A maximum variation sample of 43 UK patients with gout (29 men; 14 women; age range 32–87 years) were recruited from general practices, rheumatology clinics, gout support groups and through online advertising.

**Results:**

Severe joint pain, combined with no obvious signs of physical trauma or knowledge of injury, caused confusion for patients attempting to interpret their symptoms. Reasons for delayed consultation included self-diagnosis and/or self-medication, reluctance to seek medical attention, and financial/work pressures. Factors potentially contributing to delayed diagnosis after consultation included reported misdiagnosis, attacks in joints other than the first metatarsophalangeal joint, and female gender. The limitations in using serum uric acid (SUA) levels for diagnostic purposes were not always communicated effectively to patients, and led to uncertainty and lack of confidence in the accuracy of the diagnosis. Resistance to the diagnosis occurred in response to patients’ beliefs about the causes of gout and characteristics of the people likely to be affected. Diagnosis prompted actions, such as changes in diet, and evidence was found of self-monitoring of SUA levels.

**Conclusions:**

This study is the first to report data specifically about patients’ pathways to initial consultation and subsequent experiences of gout diagnosis. A more targeted approach to information provision at diagnosis would improve patients’ experiences.

**Strengths and limitations of this study**

- This study is the first to focus specifically on patients’ experiences of gout diagnosis, including their decisions about how and when to first seek medical advice. A sample of people from different social backgrounds and age groups from across the UK were interviewed.
- Patients’ accounts and understandings of what healthcare professionals did and said are, of course, their own perceptions. Accounts of the patient experience are of high importance in the current patient-centred provision of healthcare.
- Data presented are based on patients’ retrospective accounts of their experiences, which may change with time. In addition, some patients’ accounts of diagnosis and interactions with healthcare professionals in the past years may reflect issues or ideas that are no longer prevalent in current practice. However, the sample also included patients diagnosed recently, implying that the study's findings can be viewed as applicable to routine practice.
- A maximum variation sample is not intended to be numerically representative, but instead allows in-depth exploration and insight into previously hidden experiences of patients with gout, including those who fit the ‘typical’ patient with gout profile and those who do not. Consequently, the use of relative frequencies is avoided in the text to avoid confusion.

## Introduction

Gout is the most common form of inflammatory arthritis and affects around 2.5% of adults in the UK.^1^ Clinical diagnosis is straightforward when classical features such as sudden onset of severe joint pain (reaching peak intensity within 12–24 h), swelling, tenderness and erythema affecting the first metatarsophalangeal (MTP) joint are present.^2–8^ When these features are not present or joints other than the first MTP joint are affected, definitive diagnosis requires confirmation of the presence of monosodium urate (MSU) crystals in synovial fluid or tophi.^6^ However, the skills to perform diagnostic joint aspiration are often lacking in primary care,^9^ ^10^ where the majority of patients with gout are diagnosed and managed.^11^ This can lead to various problems with diagnosis, including misdiagnosis, delayed diagnosis, or underdiagnosis or overdiagnosis,^11–18^ particularly in females,^19^ among whom gout is less common.^1^

Much evidence has already been published documenting and measuring the extent of suboptimal management of gout and the accuracy of diagnosis.^1^ ^3^ ^13^ ^14^ ^18^ ^20–24^ Studies have focused on the quality of gout management for diagnosed patients,^14^ ^18^ ^21^ ^25^ but their emphasis has been primarily on treatment and monitoring. Those articles that have considered diagnosis have generally concentrated on diagnostic methods or accuracy.^22–24^ However, this quantitative research cannot capture patients’ stories or provide an in-depth understanding of a patient's experience and perspectives. To date, there is limited gout research using qualitative methodology. One qualitative study focused on patients’ experiences in gout management,^26^ but did offer some insight into patients’ experiences of diagnosis. A reluctance to seek advice for symptoms was reported. Negative connotations and perceptions of gout as a man's disease also meant that some patients were initially unwilling to accept the diagnosis and/or to use the term ‘gout’ to refer to their condition.^26^ Another qualitative study found that patients had ‘vivid recollections’ of their first attacks of gout.^10^

There has been no previously published research specifically examining patient experiences of gout diagnosis. Researching diagnosis is important for understanding lay experience of illness, patient compliance, health education, and other aspects of health and illness, as well as for improving non-biomedical aspects of clinical practice.^27^ ^28^ This study aimed to explore patients’ pathways to consultation and to describe key features of their experiences of gout diagnosis.

## Research methods

### Design

This study aimed to explore patients’ pathways to gout diagnosis and to capture their narratives of going through this process. A qualitative design was used to gain a greater understanding of patient experiences, and to identify key features and issues within these experiences.

Informed written consent was obtained from all participants.

### Sampling and recruitment

Maximum variation sampling (purposively selecting a heterogeneous sample)^29^ was used to select the sample. The rationale behind this method was that ‘common patterns that emerge from great variation are of particular interest and value in capturing the core experiences and central, shared aspects or impacts’ of gout diagnosis.^29^ A wide range of recruitment methods was employed, including the use of recruitment posters in general practices and rheumatology clinics, information packs handed out by members of the study advisory group, information put on local and national gout support group websites, and other online advertising sites. Interview recordings were also intended for use as an online resource about experiences of gout (healthtalk.org); thus, a wide geographical spread of participants was required to provide representation of people across England, Wales and Scotland. A literature review, and a clinical and user advisory group were used to draw up a list of other categories (in addition to geographic region) that covered the types of experiences and demographic variables that were considered to be of most importance to clinicians and patients. Potential participants who responded quickly to recruitment advertisements were used to fill the most common categories (current age, age at diagnosis, sex and years since gout diagnosis), with the only inclusion criteria being a self-reported diagnosis of gout and a minimum age >18 years. As the study progressed, recruitment was targeted by selective recruitment of people who responded to study advertisements (according to individual characteristics) to ensure that all other categories (nationality, geographical location, marital status, living arrangements, work status, number of attacks in past 12 months) were covered. Ongoing recruitment was discussed by the research team and when 43 individuals had been interviewed, it was agreed that a point of data saturation had been reached and that the sample was sufficient to provide representation across all categories.

### Data collection

Face-to-face individual semistructured interviews with 43 participants were conducted by an experienced qualitative researcher (JL). Participants were contacted by telephone or email to fix the interviews. All interviews took place in the participants’ homes or workplaces. Following informed consent, interviews were audio or video recorded depending on the participant's preference. A narrative approach^30^ was used to encourage individuals to talk about living with gout and their experiences. This facilitated the exploration of each individual's own concerns, meanings and priorities related to gout diagnosis, rather than these being imposed by predetermined questions.^31^ The researcher used an interview guide to explore areas in more depth, and to ask about topics that had not been previously mentioned.

### Data analysis

Interview recordings were transcribed verbatim and checked by the researcher (JL). NVivo V.9 (QSR) computer software was used to facilitate data coding, sorting and retrieval. JL read and re-read all transcripts and constructed a coding frame of themes using the method of constant comparison.^32^ A second researcher (SP) checked these to identify any additional codes. Analytic themes relating to diagnosis were then discussed and developed further by JL and JCR,^33^ and existing models of delays to diagnosis and pathways to treatment were used as frameworks to assist in the analysis of the process leading to diagnosis.^34^ ^35^ Further analyses and extracts from interviews are presented on http://www.healthtalk.org and illustrated with interview excerpts.

## Results

Quotations are used to represent the range of responses expressed by participants and illustrate the findings. All names used are pseudonyms.

### Sociodemographic characteristics

The sample included people from different age groups, social backgrounds and geographical areas, people from two ethnic groups, and men and women who had been diagnosed recently as well as those who had lived with gout for many years (table 1).

**Table 1:** Sociodemographic characteristics of sample

	Men (%) (n=29)	Women (%) (n=14)	Total (%) (n=43)
Age group at interview (years)			
30–49	4 (14)	3 (21)	7 (16)
50–69	16 (56)	7 (50)	23 (54)
70–89	9 (31)	4 (28)	13 (30)
Age group at diagnosis (years)			
<30	1 (3)	1 (7)	2 (5)
30–49	19 (66)	4 (29)	23 (53)
50–69	9 (31)	7 (50)	16 (37)
70–89	–	2 (14)	2 (5)
Time since diagnosis (years)			
1–5	4 (14)	6 (43)	10 (23)
6–10	2 (7)	4 (29)	6 (14)
11–15	6 (21)	3 (21)	9 (21)
≥16	17 (59)	1 (7)	18 (42)
Ethnicity/nationality			
White British	27 (93)	13 (92)	40 (92)
Asian British	2 (7)	1 (7)	3 (7)
Geographical location			
England	28 (97)	11 (79)	39 (91)
Scotland	–	2 (14)	2 (5)
Wales	1 (3)	1 (7)	2 (5)
Living arrangements			
Living alone	2 (7)	4 (29)	6 (14)
Living with one other person	20 (69)	6 (43)	26 (60)
Living with more than one other person	7 (24)	4 (29)	11 (26)
Marital status			
Married/long-term partner	27 (93)	9 (64)	36 (84)
Single	–	2 (14)	2 (5)
Divorced/separated	–	2 (14)	2 (5)
Widowed	2 (7)	1 (7)	3 (7)
Current work status			
Retired	16 (55)	9 (64)	25 (58)
Full-time work	6 (21)	5 (36)	11 (26)
Part time work	5 (17)	–	5 (12)
Student (higher education)	1 (3)	–	1 (2)
Not working for health reasons	1 (3)	–	1 (2)
Attacks in past 12 months			
0	10 (3)	4 (29)	14 (33)
1–4	16 (55)	6 (43)	22 (51)
5–9	1 (3)	1 (7)	2 (5)
≥10	2 (7)	3 (21)	5 (12)
Recruitment source			
Newspaper/magazine/newsletter/email advertisement	5 (17)	4 (29)	9 (21)
Research team colleagues/personal contacts	3 (10)	1 (7)	4 (9)
Advertisement seen by participant's friend/colleague	6 (21)	5 (36)	11 (26)
Local radio	3 (10)	1 (7)	4 (9)
Online gout forum/website	3 (10)	2 (14)	5 (12)
Patient group	4 (14)	–	4 (9)
Health professional	2 (7)	1 (7)	3 (7)
Internet search	1 (3)	–	1 (2)
More than one source (newspaper and radio)	1 (3)	–	1 (2)
Unknown	1 (3)	–	1 (2)

### Overall findings

Experiences consistent with a typical onset of symptoms featured strongly in patients’ accounts—they woke up during the night or in the morning with severe pain^36^ ^37^ which was often, though not exclusively, in the first metatarsophalangeal (MTP) joint.^2–5^ Patients described a distinctive pain, unlike any other they had experienced before.

My left knee was in complete agony. I couldn't bend it. […] it was not like any sort of pain I’d had before. It wasn’t particularly swollen. […] there was no obvious signs of injury. It was very debilitating because basically I couldn’t move it at all. (Andrew, 50yrs)

Descriptions of gout causing the worst pain patients had ever had—even compared with heart attacks and childbirth—were common. This had made their first attacks particularly memorable; therefore they were able to give rich, detailed accounts of their gout pain experiences.

A common belief among patients who described a clinical diagnosis was that the diagnosis was made based on blood tests to measure serum uric acid (SUA) levels, despite the fact that these tests cannot confirm or exclude a diagnosis of gout.^5^ ^6^ Wendy described how blood samples were taken, and the diagnosis was made when she returned for the results of the SUA test:

So I got myself off to the doctors and that's what he said, ‘oh yes it is red and it's hot and, you know, I think you've got gout’. But he said we won't know for sure until we've done these blood tests. (Wendy, 66yrs).

### Main themes in diagnosis

The analysis resulted in six main themes related to diagnosis. Key points within each theme are summarised below. Online supplementary material, including supporting data and further details of each theme, is available at *BMJ Open* online.

#### Patients’ interpretations of symptoms

Patients described how, once they experienced pain symptoms, they began to analyse these to try to identify the cause and determine what action to take. Patients considered a wide variety of possible causes for their symptoms, including infection, insect bites, chilblains, working too hard and broken toes. The intensity of pain was sometimes particularly worrying because patients thought it could be caused by a life-threatening illness. The location of pain within and/or around a joint was a factor that appeared to cause particular confusion. The location, combined with the severity of the pain, led patients to believe that the pain could be a result of physical trauma, but this conflicted with their knowledge that they had not injured themselves and could not see any obvious signs of physical trauma.

#### Decisions about seeking medical attention

Patients’ approaches to deciding *if* or *when* to consult a medical professional ranged widely, from those who made an appointment to see the general practitioner (GP) as soon as possible—often motivated by pain intensity—to those who waited for several months or years before seeking advice. Delays in seeking medical advice were due to factors such as self-diagnosis/treatment, financial pressures, and being on holiday abroad at the time of a first attack.

I'd worked with it for 3 weeks, walking on the side of my foot […] it's probably the worst pain I've had, in my life […] I was a piece worker so what I made I got paid for, if I didn't make it, I didn't get paid, so and, you know, we were, young family then, so… […] eventually I just had to give in and go to the Doctors and get signed off for a week or two. (Steve, 64yrs)

#### Triggers and delays within the diagnostic interval

The ‘diagnostic interval’ describes the period of time between the first appointment with a healthcare professional and the formal diagnosis being made.^35^ This varied widely, from those who were diagnosed in their first appointment or a few weeks later to those who were not diagnosed until a later appointment, several months or years later.

I went to the A&E […] I saw a doctor there and he thought I'd been bitten on the foot, which was interesting, and they gave me some drawing paste to put over the actual area, it was the usual place, on the big toe, swollen there, and he said, ‘If it doesn't improve come back and we'll have a look at it’. […] Obviously it didn't improve […]The doctor didn't identify what it was […] it was just really a shot in the dark, so my medical advice then was not geared towards gout. (Henry, 63yrs)

Patients talked about a number of delays and problems they encountered during the period before a diagnosis was made, including misdiagnosis and difficulty getting an appointment when symptoms were most severe. Some patients with gout attacks in joints other than the first MTP joint, such as the knee, had their symptoms attributed to other causes such as sports injuries. Gender was another factor that appeared to cause delays for some women.

#### Accepting or doubting the diagnosis

Patients who remained unconvinced about the accuracy of the diagnosis either questioned the severity of their symptoms, the method of diagnosis, or had not reconciled the diagnosis with their ideas about the causes and characteristics of people likely to be affected.

I often doubt, I think ‘oh have they got it wrong?’ you know, because I don't speak to anybody who’s got it, especially my age, you’re talking like really—much older men, like my Nanna knows people who’ve got it, but […] they’re not women. (Georgina, 41yrs)

Others felt that their diagnosis of gout was more the result of a process of elimination than it was a process of identification (or confirmation) of the condition, leading them to suspect that doctors were ‘not sure’ about what was causing their symptoms.

#### Thoughts and feelings on receiving the diagnosis

A desire for greater information provision at the point of diagnosis was a strong theme in the interviews. Not all patients were aware that gout was a chronic condition that required long-term management.

While some patients were not surprised by the diagnosis, others were shocked, angry or confused because they believed their lifestyles were very different to those they associated with gout. The contradiction was reinforced by some health professionals placing an emphasis on lifestyle changes. Resistance to the label of ‘gout’ featured strongly in some interviews, and some interviewees chose to explain their symptoms as ‘arthritis’, rather than as gout, to other people.

I think probably in the beginning, I didn't actually tell anyone. I just said, ‘Oh, I had a pain. I had trouble with my big toe.’ […] I think because of that sort of possibly kind of embarrassment. It's easier to say that you've got arthritis or something, rather than saying that you've got gout. […] Probably because people might assume that you drink and eat lots of red meat and you're overweight and things like that, and you're not. (Judith, 61yrs)

#### Actions on receiving the diagnosis

Being diagnosed with gout was an immediate prompt for further action by some patients. For example, they spent time searching the internet for information about the condition, joined internet forums or discussion groups for people with gout, or asked their GP for referral to a specialist.

One key unanticipated finding that has not previously been reported in the literature was that, after doing research on the internet following diagnosis, two patients had bought equipment to monitor their own SUA levels.

I've got one of those little kits that you use. So I wanted to make sure that […] wanted to understand my own disease and manage my own disease, so I've been doing that ever since. […] So, the first thing I did was, I calibrated my monitor with the blood test. […] that seemed like an accurate representation of what the bloods were telling me. […] And I've actually, at some points, titrated my own dose of Allopurinol. (Adam, 41yrs)

Another common pattern was that patients began to change their diets in response to information they read on the internet, regardless of whether or not their GP had recommended such changes.

## Discussion

This paper is the first to look specifically and in-depth at patients’ pathways to initial consultation and experiences of gout diagnosis. Several issues were raised by patients that may have led to delays in their diagnoses. Owing to its qualitative nature, the study also resulted in unanticipated findings^31^ around self-monitoring. It is not appropriate to draw inferences about the prevalence of experiences from these qualitative data.^38^ However, these do provide an insight into the nature and range of issues that patients may experience in the diagnosis of gout. This has implications for clinical practice in terms of understanding and addressing delays in the diagnostic interval, and improving patient experiences of gout diagnosis.

### Understanding delays in diagnosis

Consistent with other descriptions of processes leading to diagnosis,^34^ ^35^ patients analysed their symptoms to try and identify the cause and to determine what action to take. Evidence about patient decisions of *how* and *when* to seek medical advice—particularly the fact that some patients waited months or even years before consulting—contributes to the understanding of why gout is sometimes diagnosed late in its clinical course.^9^ ^10^ Links between the reluctance to seek medical attention and patients’ embarrassment of admitting to the pain of gout have previously been reported,^26^ but the data here suggest that other factors such as financial pressures, first attacks occurring during trips outside the UK, self-diagnosis and/or self-medication may also play a part. Additional factors that may have caused delays (during the diagnostic interval) for the patients in this study included misdiagnosis, attacks in joints other than the first MTP joint, female gender, and consultations taking place after the period when symptoms were at their peak intensity (when visible classical features of an attack may have been less evident). These findings are useful in identifying the range of factors that may contribute to delayed diagnosis, and perhaps require further exploration. In particular, it would be useful to explore experiences of diagnosis with patients from a wider range of ethnic backgrounds in order to ascertain whether there are cultural factors contributing to delayed diagnosis in some cases.

### Implications for self-management

One unanticipated finding from the study is that diagnosis prompted several patients to proactively attempt to take control of managing their condition by purchasing equipment to self-monitor SUA levels. Given that this finding has not been widely reported before, clinicians should be aware that patients may be monitoring their own SUA levels and may wish to discuss these. In addition, this finding may be relevant for future studies looking into the acceptability of self-monitoring or self-titration.

### Targeting information provision at diagnosis to improve patients’ understanding and confidence

From the findings reported in this study, several key areas can be identified where a more targeted approach to information provision at diagnosis would improve patients’ experiences. These are relevant both for information provision in the consultation, and for decisions about the information that is included in gout patient information leaflets, websites or other materials.

The first issue is around the measurement of SUA levels. Despite the fact that the results of SUA tests cannot confirm or exclude a diagnosis of gout,^5^ ^6^ findings from this study suggest that the limitations in using SUA levels for diagnostic purposes are not always (effectively) communicated to patients. Understanding gout has previously been stated as important for patient adherence and self-management;^11^ ^39^ ^40^ so improving patient understanding of this aspect of gout would be beneficial.

The second key point is that male and female patients may be resistant to being labelled with a diagnosis of gout. Negative views about gout can impact on disease management and adherence to urate-lowering therapy (ULT).^41–43^ Emphasising that gout is a form of arthritis may give patients greater confidence in accepting the diagnosis and communicating it to others. In addition, reassurance could be offered to female patients that, although less common, it is not unusual for women to have gout. Research with patients from a wider range of ethnic groups may also provide insights into providing information to reflect any cultural differences in the understanding or the experiences of gout.

The third issue centres around raising awareness of gout as a chronic condition that requires long-term management.^12^ ^39^ Patients leaving the consultation thinking of gout as an intermittent, acute condition, clearly has implications for their subsequent behaviour and perceptions of the need for ULT. If patients are not aware that long-term management is important, they may be less likely to return if they experience subsequent attacks, relying instead on self-medication.

The fourth key finding is that getting an optimal balance of information provision around lifestyle factors^12^ may be difficult. Too great an emphasis, as perceived by patients, was seen as judgemental, and reinforced misconceptions about the most common causes of gout and concerns about the condition being perceived as self-inflicted. On the other hand, perceiving that they were not given enough information could leave patients feeling ‘fobbed off’ and/or making drastic or unhealthy changes to their diets. The other challenge is for clinicians to balance information about non-pharmacological approaches (which may initially be prioritised by patients), and emphasis on more effective (but potentially less acceptable to some patients) pharmacological approaches.^11^ ^12^ In addition, some patients seemed prepared to accept information they found on the internet despite the fact that this conflicted with advice from their GP. Previous research has also found that some patients value online resources more than doctors as information sources.^44^ Future research could usefully explore these issues further in the context of gout diagnosis.

Focusing on these key areas (reasons for measuring SUA levels, gout as a form of arthritis, the chronic nature of gout, and the links between gout and lifestyle factors) in the consultation would increase confidence in the diagnosis and treatment, and help to address misconceptions about the causes of gout and the characteristics of people who are diagnosed with it.

### Strengths and limitations

This paper is the first to focus specifically on patients’ experiences of gout diagnosis, including their decisions about how and when to first seek medical advice. The sample of people from different social backgrounds and age groups allowed for in-depth exploration and insight into previously hidden experiences of patients with gout, including those who fit the ‘typical’ gout profile and those who do not. The study included patients from two ethnic groups, but it would be useful to explore experiences of diagnosis with patients from a wider range of ethnic backgrounds in order to ascertain whether there are cultural factors impacting on experiences of diagnosis in some cases. In addition, while accounts of patients’ experience are of high importance in achieving patient-centred provision of healthcare, patients’ accounts and understandings of what health professionals *did* and *said* are, of course, their own perceptions. Retrospective accounts of experiences may also change with time, or reflect issues or ideas that are no longer prevalent in current practice.

## Conclusion

This study is the first to report data specifically about patients’ pathways to initial consultation and subsequent experiences of gout diagnosis. These data provide insight into the nature and range of issues that patients may experience in the diagnosis of gout. Such evidence has implications for clinical practice in terms of understanding and addressing delays in diagnosis, and adopting a more targeted approach to information provision at diagnosis to improve patients’ experiences.
